# Orthogonal ensemble and single-particle characterisation of antisense oligonucleotide lipid nanoparticles

**DOI:** 10.1039/d6pm00312e

**Published:** 2026-11-05

**Authors:** Callum G. Davidson, Pascale Rae, Savvas Ioannou, Yvonne Perrie, Zahra Rattray

**Affiliations:** a Strathclyde Institute of Pharmacy and Biomedical Sciences, University of Strathclyde 161 Cathedral St. Glasgow G4 0RE Scotland UK zahra.rattray@strath.ac.uk; b School of Molecular Biosciences, University of Glasgow Glasgow G11 6EW UK

## Abstract

Antisense oligonucleotide (ASO) lipid nanoparticles (LNPs) remain underexplored relative to siRNA and mRNA LNPs. Using an orthogonal workflow (DLS, NTA, AF4), we show that ionisable lipid identity and microfluidic flow rate govern key LNP attributes. Replacing SM-102 with MC3 increased size and reduced ASO recovery, while lower flow rates increased LNP size and improved recovery, highlighting formulation–property relationships for ASO LNP optimisation.

## Abstract 2

A stepwise optimisation strategy for ASO-loaded LNPs integrating microfluidic LNP manufacture with high-resolution CQA analysis, enables data-driven formulation refinement, and accelerates LNP design.

## Graphical abstract

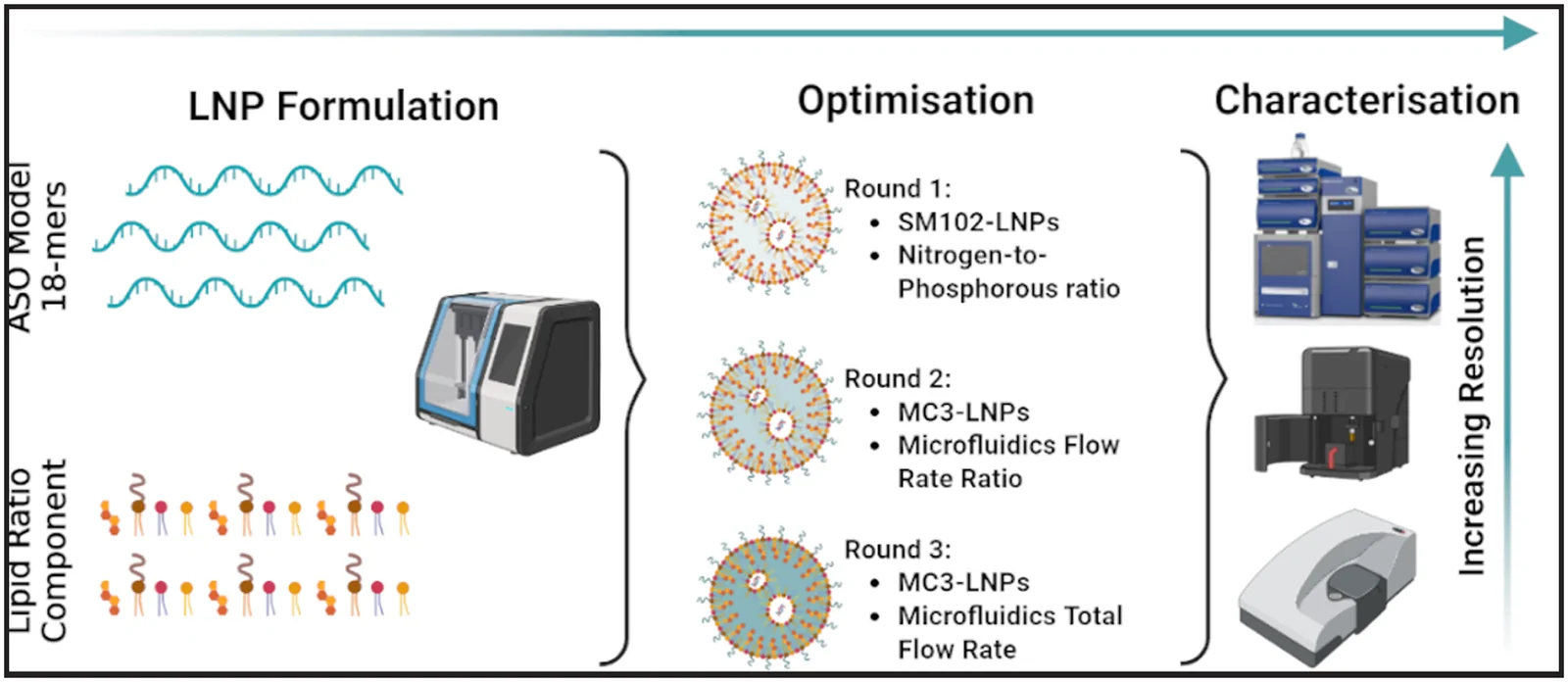

Oligonucleotide (ON) therapeutics have emerged as a powerful modality for modulating gene expression. Among these, antisense oligonucleotides (ASOs) enable targeted regulation of protein expression *via* binding to complementary messenger RNA (mRNA), promoting RNAse-mediated degradation or modulating splicing to alter protein translation.^1^

ASOs face significant biological barriers to their therapeutic efficacy, including rapid enzymatic degradation, renal clearance and extensive blood–serum protein binding, and inherently poor cellular uptake due to their polyanionic, hydrophilic nature. While chemical modifications can enhance nuclease resistance and binding affinity, efficient delivery remains a major limitation.^2–4^ Lipid nanoparticles (LNPs) offer a promising strategy to enhance stability and facilitate intracellular delivery. Whilst LNPs have been extensively studied for the delivery of siRNA and mRNA payloads,^5–7^ the encapsulation of single-stranded ASOs within LNPs remains under characterised.^8–14^ Orthogonal analytical approaches provide complementary insight into complex nanomedicine formulations, enabling detailed physicochemical characterisation and informed optimisation of formulation candidates, using low- and high-resolution analytical techniques.^15,16^

Herein, LNPs encapsulating an 18-mer Nusinersen analogue were prepared *via* microfluidic mixing (Table S3). The influence of key LNP formulation parameters, including nitrogen-to-phosphate (N/P) ratio, ionisable lipid composition, flow rate ratio (FRR), and total flow rate (TFR) on physicochemical properties was systematically investigated. LNP-1 and LNP-2 were formulated using the ionisable lipid SM102 at N/P ratios of 6 and 12, respectively. LNP-3–LNP-5 contained MC3 as the ionisable lipid and were used to evaluate formulation parameters, including a comparison of FRR of 3 (LNP-3) and 6 (LNP-4), and TFR of 15 mL min^−1^ (LNP-3) and 5 mL min^−1^ (LNP-5). Additionally, the impact of ionisable lipid chemistry was assessed by comparing SM102-based LNP-1 with MC3-based LNP-3, which were prepared using equivalent formulation and purification conditions.

Comparison of LNP size distributions (Fig. 1) shows that LNP-1 produced the smallest particle sizes, and broadest size distributions (PDI ∼ 0.14). In contrast, LNP formulations 2–5 did not follow a consistent size–distribution relationship such that increases in particle size were accompanied by narrower size distributions (Fig. 1 and Tables S5, S6). The selection of ionisable lipid had a pronounced effect, with LNP-1 (SM102) and LNP-3 (MC3) formulations having the largest size and PDI/span differences under equivalent manufacturing conditions (Fig. 1 and Tables S5, S6).

**Fig. 1:**
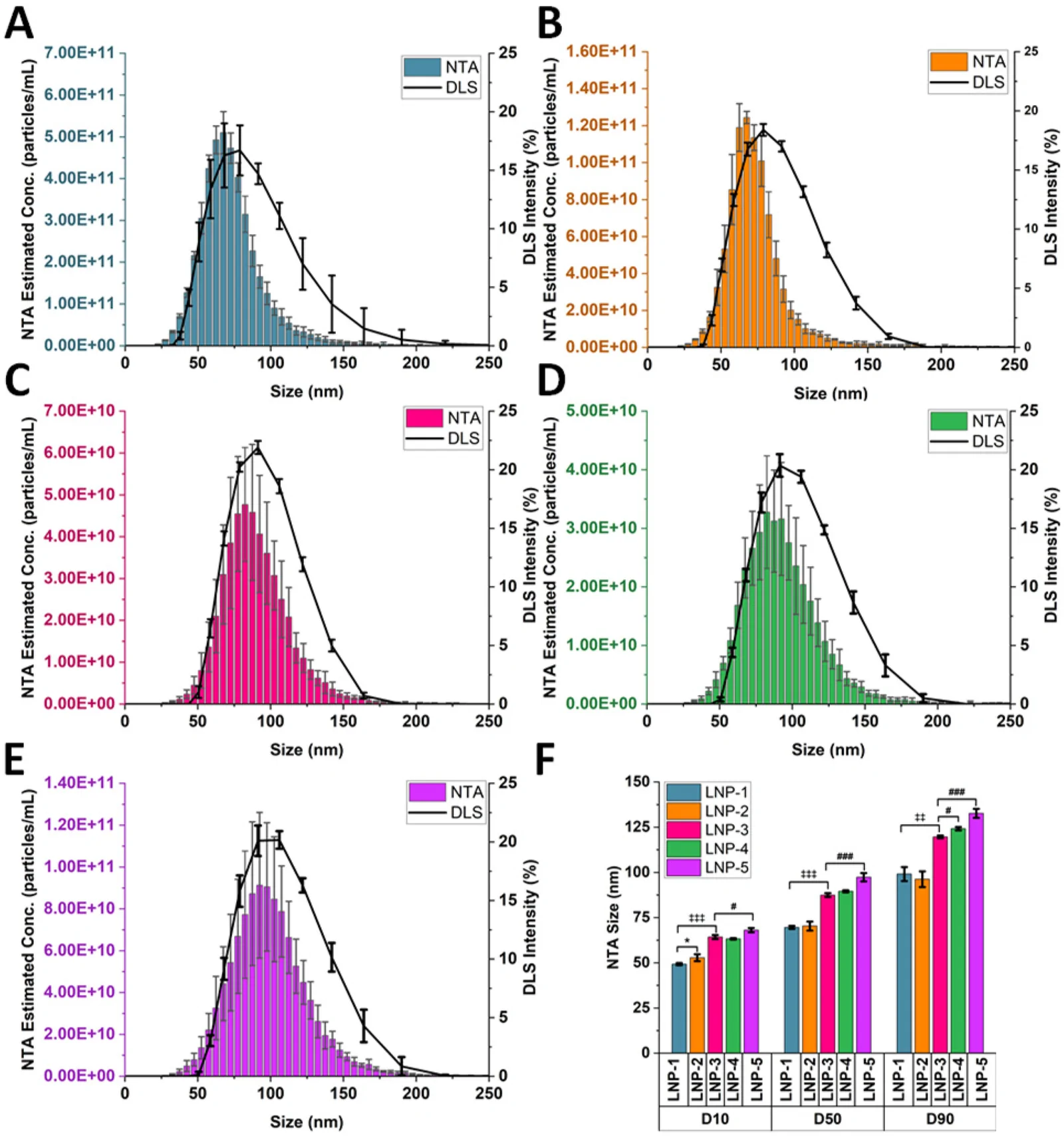
Physicochemical properties of ASO-LNPs comparing DLS and NTA size distributions. (A) LNP-1; (B) LNP-2; (C) LNP-3 (D) LNP-4; (E) LNP-5; and (F) NTA *D*_10_, *D*_50_, *D*_90_ distributional percentile values. (*n* = 3 ± STD). Tukey tests compared LNP-1–2 (*), LNP1–3(‡) and LNP3–4/5(#) where *p < 0.05, **p < 0.005 and ***p < 0.0005.

To further quantify LNP size and polydispersity at higher resolution, NTA was used. Formulation-dependent differences were observed, particularly in particle concentration (Fig. 1). LNP-1 exhibited the highest concentration (5 × 10^11^ particles per mL), followed by LNP-2 (1.2 × 10^11^), LNP-5 (9 × 10^10^), and LNP-3 and LNP-4 (∼5 × 10^10^). These variations highlight formulation-dependent differences in recovery following spin column purification, with LNP-1 yielding approximately 10-fold higher particle concentrations than LNP-3/4 (Fig. 1). Notable differences in particle diameter were also evident between LNP formulations, consistent with DLS intensity-based size measurement data (Fig. 1 and Table S5). Overall, modulation of LNP formulation parameters resulted in changes in *Z*-average and cumulative particle sizes, which increased with higher N/P ratio, an increase in FRR, decrease in TFR and change in ionisable lipid. Increasing the N/P ratio (LNP-1 *vs.* LNP-2) improved monodispersity, whereas increasing total lipid content had minimal impact on particle size, ζ-potential, and encapsulation efficiency.

Switching the ionisable lipid from SM102 to MC3 (LNP-1 *versus* LNP-3) resulted in a larger particle size, reduced size uniformity, a more negative zeta potential and lower mass balance for LNP-3 (Table S5). Increasing the FRR from 3 to 6 (LNP-3 *versus* LNP-4) produced a modest increase in particle size, potentially reflecting enhanced RNA incorporation or a more dilute lipid phase favouring formation of larger LNPs, while other physicochemical properties remained comparable (Table S5). Reducing the TFR from 15 to 5 mL min^−1^ (LNP-3 *vs.* LNP-5) further increased particle size (∼10 nm), with PDI remaining low and ζ-potential and encapsulation efficiency and mass balances were largely unchanged (Table S5). The small mean-mode differences suggest that spin-column purification yields more uniform populations. Further work would investigate pre-purification size distributions to determine if uniformity is formulation-dependent, or results from the removal of smaller-sized particles during centrifugation with spin-columns. These observations were further supported by the span values (Table S6), which followed the same trends as PDI data. LNP-1 exhibited the broadest distribution (0.72), whereas LNP-2–5 showed narrower spans (0.62–0.68). Mode diameters increased progressively across formulations by up to ∼30 nm, with LNP-5 displaying the largest particles. Distribution metrics (Fig. 1 and Table S6) further highlight the influence of formulation and processing parameters on LNP sub-populations, with *D*_10_, *D*_50_, and *D*_90_ values increasing systematically with parameter variation.

To enable further in-depth analysis of the ASO LNP formulation compositional variability, asymmetric flow field flow fractionation (AF4) was used to provide size-based separation of LNP particles based on their hydrodynamic diameters, which are governed by their diffusion in the channel flow. Unlike NTA, AF4 physically separates particles prior to detection, minimising coincident scattering effects and enhancing resolution for polydisperse systems. In this study, frit inlet AF4 (FI-AF4) was multiplexed with UV detection (260 nm) for RNA quantification, multiangle light scattering (MALS) for measuring particle radius of gyration (*R*_G_) and molar mass approximation, and online DLS, for the measurement of hydrodynamic radius (*R*_H_). Combined MALS and DLS, enabled estimation of the shape factor (*R*_G_/*R*_H_), providing insights into LNP formulation morphology and inherent structural variations between formulations.

The LNP-1 formulation consistently produced the smallest LNP size values and lowest shape factor ratio (∼0.6), deviating from the value expected for a homogeneous sphere (0.775), indicative of a dense core–shell morphology (Fig. 2).^17^ In contrast, LNP2–5 formulations exhibited larger *R*_G_ and *R*_H_, and shape factor values, with shape factors characteristic of more spherical structures. Further analysis of the LNP formulation distribution indicates larger measured particle size within LNP 2–5 formulations across 10, 50 and 90 centiles. However, this increase in particle size did not directly correlate with shape factor values, suggesting that changes in formulation parameters influence both particle growth and internal structural organisation independently (Fig. 3 and Table S7).

**Fig. 2:**
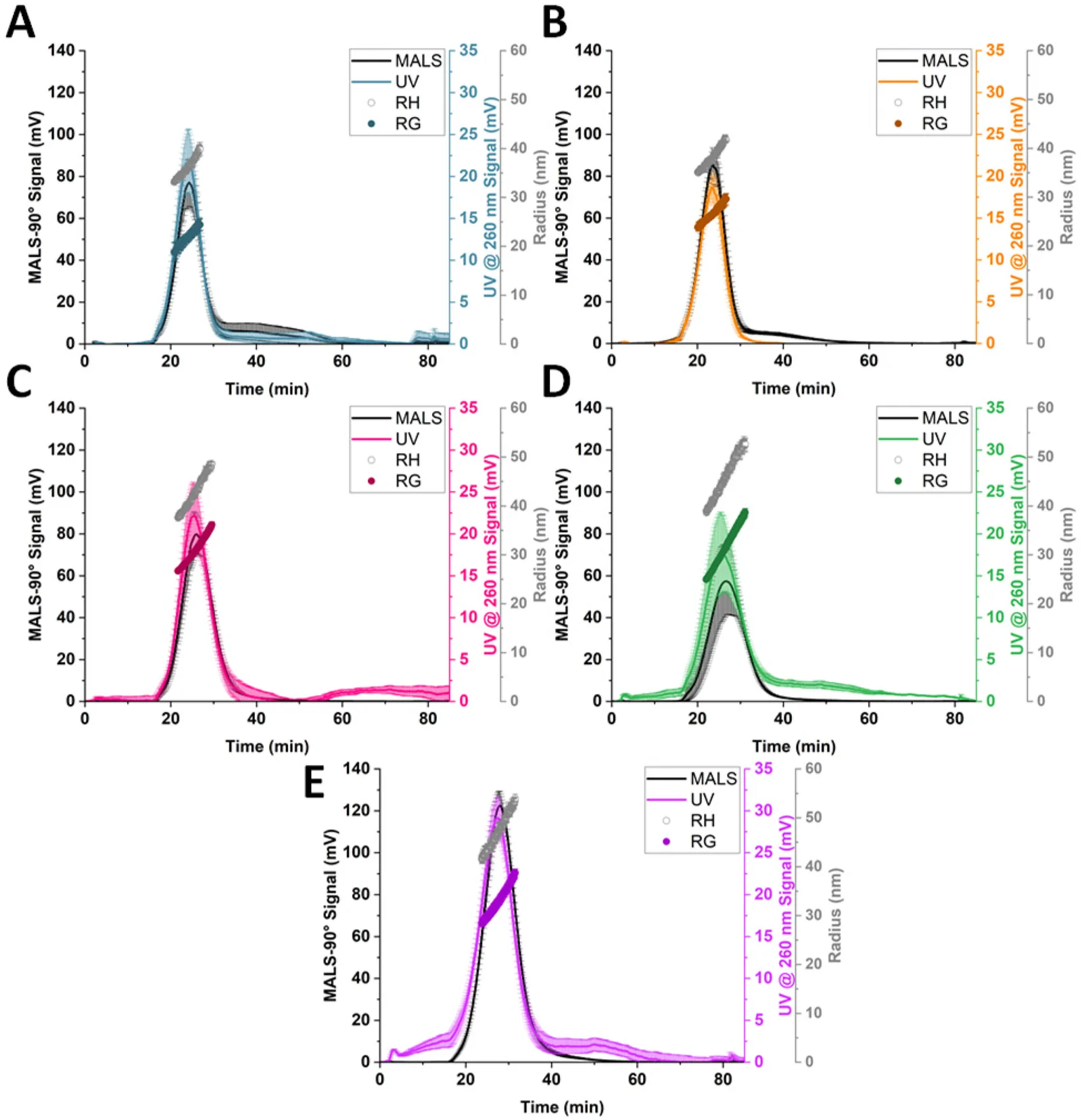
FI-AF4 fractogram elution profiles showing MALS-90° and UV260 nm traces, overlaid with main peak FWHM radius of gyration (*R*_*G*_) and hydrodynamic radius (*R*_*H*_) values. (A) LNP-1; (B) LNP-2; (C) LNP-3 (D) LNP-4; and (E) LNP-5, (*n* = 3 ± STD).

**Fig. 3:**
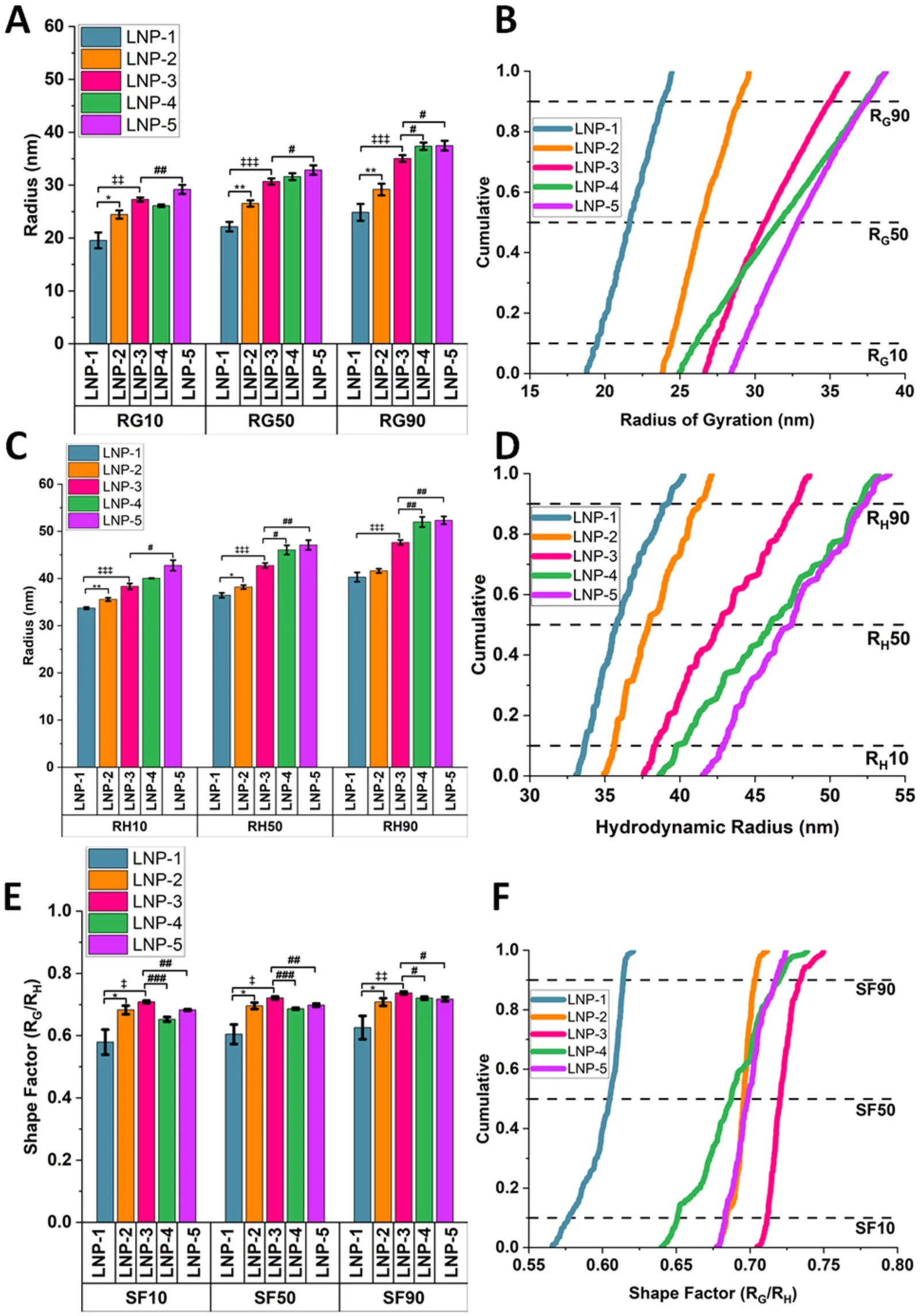
FI-AF4-UV-MALS-DLS distribution values for FWHM integrated elution profiles for ASO-LNP formulations. (A) *R*_G_ distribution (B) *R*_H_ distribution, (C) shape factor distribution. (D) *R*_G_ distribution values, factor (E) *R*_H_ distribution values and (F) shape factor distribution values. Tukey tests compared LNP1–2(*), LNP1–3(‡) and LNP3–4/5(#) where *p < 0.05, **p < 0.005 and ***p < 0.0005, (*n* = 3), error bars represent mean ± STD.

ASO therapeutics have garnered increased research interest due to their modular platform manufacture approaches and broad applicability across different novel disease targets. Clinically, ASOs are administered in their unconjugated pristine form, or are conjugated to GalNAc, a triglyceride moiety used for hepatic targeting.^18–20^ While LNPs have emerged as a popular choice for mRNA vaccines and siRNA payload delivery, their application in ASO payload encapsulation remains under reported in the literature. In this study, an orthogonal pipeline approach was used to characterise ASO-LNP physicochemical properties across a range of formulation conditions in Table S3.

Initial DLS measurements revealed monomodal LNP particle size distributions across all formulations (Fig. 1), with SM102-LNPs producing smaller hydrodynamic diameters than MC3-LNPs. Low PDI values (Table S5) indicated uniformly-dispersed particle populations and demonstrated robust microfluidic manufacture and spin-column purification. High reproducibility was reflected through small standard deviations across size and PDI values (Table S5). All LNP formulations had negative zeta potential, indicating colloidal stability defined by DLVO theory.^21^ Encapsulation efficiencies were >97% for all formulations, indicating effective incorporation and retention of ASOs during formulation (Table S5). However, ASO mass balance recovery remained relatively (∼52% for LNP-1/2 and ∼40% for LNP-3–5), indicating high losses of ASOs across formulation and purification (Table S5). These loses could be attributed to smaller LNPs being filtered during purification, the size of ASOs (18-mer) payloads having no recorded secondary interactions to further enhance singular RNA interaction with ionisable lipids during formulation, which would impact encapsulation and further recovery.

Enhancing physicochemical property resolution beyond DLS, NTA was employed to analyse LNP populations on a particle-by-particle basis, with particles size derived from diffusion coefficient using the Stokes–Einstein equation. These enhanced NTA insights showcase similar trends noted with DLS with increasing particle sizes across tested LNP formulations (Fig. 1 and Table S6). Initial particle concentration differences between formulations highlight post-spin column concentration differences with LNP-1 producing the highest concentration compared to a 10-fold decrease compared to LNP-4 (Fig. 1). Comparing both formulations show that changing ionisable lipid composition and microfluidic FRR contributed to increased particle dilution and particle detection (Fig. 1). FRR impact aligns with LNPs using different sized RNA cargo.^22^ Further comparing formulations, span values remain low, highlight formulation monodispersity (Table S6) without secondary aggregate peaks (Fig. 1).

To probe ASO-LNP structure and composition in greater depth, AF4 was applied to physically separate nanoparticles based on their diffusion-governed hydrodynamic diameters (Fig. 2 and 3). AF4 addressed the limitations of NTA by eliminating coincident detection artefacts and enabling fraction specific characterisation. AF4 method performance highlighted high sample recovery (>85%), supporting reliable downstream analysis with online detectors. MALS-90° traces were integrated at FWHM where *R*_G_ and *R*_H_ values could be obtained and utilised to infer particle shape factor morphology (Fig. 3 and Table S7). Overlaid UV/MALS profiles highlighted thar ASOs co-eluted with LNPs indicating successful ASO loading and structural stability of the formulations during separation. Larger-size particle sizes eluted at later timepoints, however evaluating LNP1/2 shows a decrease of 0.5 minute elution time but a larger size of 2 nm (Table S7). This disparity could be due to the lower RNA concentrations in LNP-2 using a higher N/P ratio producing overall larger particles that are less dense than LNP-1 with differing morphologies, in alignment with shape factor values (Fig. 3 and Table S7). These differences could also be due to LNP microenvironment differences between formulations, with LNP-2 having a higher lipid content compared to LNP-1. The increased cationic lipid content in LNP2 may improve electrostatic complexation of the phosphorothioate-modified oligonucleotide, whose altered backbone chemistry can affect interactions with ionisable lipids compared with conventional phosphodiester nucleic acids. Distributional values offered deeper insights into *R*_G_ and *R*_H_ and shape factor distributions (Fig. 3 and Table S7), with similar morphologies produced between *D*_50–90_ for all formulations except for LNP-1. Integrating physicochemical outputs across orthogonal analytical techniques further strengthens our understanding of how these attributes interrelate and can be leveraged to guide rational ASO-LNP design, using lipid chemistries and lipid geometries to enable encapsulation and efficient packing of ASOs within LNPs. This study demonstrates that the choice of ionisable lipid significantly effects variable clustering, as reflected in zeta potential and size-related outputs (Fig. S2), highlighting further emphasis on surface charge as an indicator of colloidal stability within ASO-LNPs through DLVO theory,^21^ and subsequent impact of overall main ionisable lipid selection within early ASO-LNP candidate screening (Fig. 1–3). Early ASO-LNP candidates should continue to be evaluated across independent, orthogonal analytical pipelines to ensure high-quality data that informs downstream decision-making and the candidate selection process.

## Author contributions

Conceptualisation: CGD, ZR; investigation: CGD, ZR; methodology: CGD, PR, SI, ZR; analysis: CGD, SI and ZR; writing original draft: CGD and ZR; visualization: CGD; writing—reviewing and editing: CGD, YP and ZR; funding acquisition: ZR. All authors have read and agreed to the published version of the manuscript.

## Conflicts of interest

There are no conflicts to declare.

## Supplementary Material

PM-OLF-D6PM00312E-s001

## Data Availability

All data associated with this work are included in the supplementary information (SI) or can be accessed at https://doi.org/10.15129/a03daac8-0b32-4389-a7f5-e0a442002ad0. The supplementary data file contains all methods pertaining to the work reported in this manuscript, and mean parameters associated with particle sizing of ASO LNP formulations and negative staining EM images of ASO LNPs. Supplementary information is available. See DOI: https://doi.org/10.1039/d6pm00312e.
