# Supplementary material for: Orthogonal ensemble and single-particle characterisation of antisense oligonucleotide lipid nanoparticles
Source: RSC Pharm. 2026 Nov 5. Online ahead of print. doi: 10.1039/d6pm00312e (PMC13752843; doi:10.1039/d6pm00312e)
Supplement: Supplementary file 1 [file PM-OLF-D6PM00312E-s001.pdf]

Supplementary Information (SI) for RSC Pharmaceutics.
This journal is © The Royal Society of Chemistry

Orthogonal ensemble and single-particle characterisation of antisense oligonucleotide lipid nanoparticles

a. *Callum G. Davidson[a], Pascale Rae[a], Savvas Ioannu[b], Yvonne Perrie[a], Zahra Rattray*Strathclyde Institute of Pharmacy and Biomedical Sciences, University of Strathclyde, Glasgow, G4 0RE.
b. School of Molecular Biosciences, University of Glasgow, Glasgow G11 6EW

*Corresponding author: Zahra Rattray (zahra.rattray@strath.ac.uk)

Keywords: Oligonucleotide, Drug Substance, Drug Product, Lipid Nanoparticles, Metrology

Supplemental Information

Materials

ON was manufactured by Integrated DNA Technologies (Table S1). DNA/RNA free water, 10× phosphate-buffered saline (PBS) pH 7.4, sodium citrate dihydrate and Quanit-IT RiboGreen™ RNA Quantification Assay were acquired from ThermoFisher (Fisher Scientific, Leicestershire, UK) and 1N hydrochloric acid was sourced from Alfa Aesar. 8-[(2-hydroxyethyl)[6-oxo-6- 83 (undecyloxy)hexyl]amino]-octanoic acid, 1-octylnonyl ester (SM102), (6Z,9Z,28Z,31Z)-Heptatriaconta-6,9,28,31-tetraen-19-yl 4-(dimethylamino)butanoate (MC3) were purchased from BroadPharm (San Diego, CA, USA). 1,2-distearoyl-sn-glycero-3-phosphocholine (DSPC), 1,2-dimyristoyl-rac-glycero-3-methoxypolyethylene glycol-2000 (DMG-PEG 2000) were purchased from Avanti Polar Lipids 81 (Alabaster, AL, USA). Cholesterol (CHOL) and Amicon®-15 100 kDa MWCO regenerated cellulose spin columns were sourced from Sigma Aldrich (Merck, Gillingham, UK).

Table S1 *Model ON sequences and associated molecular weight.*

ON	Sequence	Molecular Weight (Da)
ON – 18-mer	5'-UCACUUUCAUAAUGCUGG-3'<br>Modifications: 2'-OMe, PS	6179.9

Methods

Drug Substance Analysis

Liquid Chromatography Hyphenated UV/Vis was used compared ion-pairing and hydrophilic interaction separation to quantify low/high molecular weight species and ON full length product. *Table S2* denotes systems and separation parameters used.

Table S2 *LC-UV separation and detection parameters for ON.*

Parameter	Value			
System	Waters ACQUITY Premier UHPLC system.		Shimadzu UHPLC (SCL-40, LC-40B-X3, SIL-40C X3, CTO-40C, SPD-M40).	
Column	Waters Premier Oligonucleotide BEH C18 (100 x 2.1 mm, 1.7 μm, part #186009485)		Waters Premier BEH Amide (150 x 2.1 mm, 1.7 μm, part #186004802)	
UV Detector	260 nm		260 nm	
Autosampler Temperature	4 °C		4 °C	
Mobile Phase A	50 mM HFIP, 5 mM N,N-diisopropylethylamine in water		20 mM Ammonium Acetate	
Mobile Phase B	50 mM HFIP, 5 mM N,N-diisopropylethylamine in (50/50, v/v) Water/Acetonitrile		Acetonitrile	
Flow Rate	0.35 mL/min		0.30 mL/min	
Injection Volume	2 μL		5 μL	
Column Temperature	40 °C		40 °C	
Gradient	Time (min)	% B	Time (min)	% B
	0	10	0	90
	10	40	45	50
	10.1	10	60	50
	11.0	100	60.1	90
	11.1	10		

Preparation of ASO-LNP Formulations by Microfluidics

LNP formulations were composed of SM102/MC3: CHOL: DSPC:DMG-PEG2000. All initial lipid stock solutions were prepared in ethanol at 5 mg/mL and combined at a 50:38.5:10:1.5 molar ratio for ionisable lipid: cholesterol:

helper:PEG-lipid at 0.6 mL. ASOs were prepared in DNAse/RNAse free water at 2 mg/mL and diluted in 50 mM citrate buffer (pH 4.0) to 1.2 mL, which was used as the aqueous phase. An Ignite+ microfluidics system was utilised for LNP synthesis using a toroidal chip architecture Lipid organic phase and ASO aqueous phases were injected simultaneously into the micromixer using parameters in Table S3. LNP formulations (1 mL) were purified using spin column centrifugation (100 kDa MWCO). Briefly LNPs were diluted (1:40) in PBS (pH 7.4) and centrifuged at 2,000 x g at 4 °C to remove residual ethanol until resultant formulation remained (1 mL).

Table S3 *ASO-LNP Formulation Optimisation design. Formulation variations are highlighted in bold.*

LNP #	Formulation Optimisation Parameter			
	Ionisable Lipid	N/P Ratio	Flow Rate Ratio	Total Flow Rate
**1**	SM102	6	3	15
**2**	SM102	**12**	3	15
**3**	**MC3**	6	3	15
**4**	MC3	6	**6**	15
**5**	MC3	6	3	**5**

Characterisation of ASO-LNP Formulation CQAs

ASO-LNPs were characterised using a Zetasizer, RiboGreen™ assay, Nanoparticle tracking analysis (NTA), frit-inlet asymmetric-flow field-flow fractionation hyphenated with multiple online detectors (FI-AF4-MD) and imaged by negative stain transmission electron microscopy.

Dynamic light scattering particle size (Z-average) and polydispersity index (PDI) were determined using a Zetasizer Nano ZS system (Malvern Panalytical, Worcestershire, UK) equipped with a 633 nm Helium-Neon laser and a detection angle of 173° (non-invasive back scattering). Material parameters were set to 1.45 refractive index and 0.001 absorption. Unless otherwise stated, all measurements were performed at 25 °C and at a 1:10 dilution in PBS (pH 7.4) for all LNP samples. All measurements were performed in three independent replicate measurements consisting of at least two technical replicates.

Electrophoretic light scattering **(ELS)** zeta potential surface charge was measured using ELS post manufacture/process test. Unless otherwise stated, all measurements were performed at 25 °C and at a 1:10 dilution in DNA/RNAse free water for all LNP samples. All ζ-potential measurements were performed using three biological replicate measurements consisting of at least two technical replicates.

RiboGreen™ assay evaluated the encapsulation efficiency (EE) and drug recovery mass balance (MB) of the ASO cargo during LNP formulation. Both EE and MB were measured using the Quant-iT™ RiboGreen™ RNA Quantitation assay Kit (Thermo Fisher #4110) as per manufacturer instructions. The kit quantifies the total RNA and unentrapped RNA on the LNP surface to calculate the efficiency of drug encapsulation during formulation. The assay also determines recovery by comparing the total formulation RNA content to a theoretical RNA concentration. Corresponding fluorescence intensity of the RiboGreen™ signal was measured on the GloMax® Explorer GM3500 microplate reader (Promega, UK) at an excitation wavelength of 475 nm, with the emitted fluorescence measured at 500-550 nm. All fluorescence data were captured at ambient temperature (25 °C) using a GloMax® firmware version 4.29.0 and processed using GloMax® Fluorescence software version 3.1.0.

Nanoparticle tracking analysis *(NTA)* was used to measure particle size, distribution, and estimated particle concentration was conducted using a NanoSight NS300 system (Malvern Panalytical, UK), equipped with a 488 nm laser, low-volume flow cell, sCMOS camera, and automated syringe driver. Samples were diluted 1:10,000 in PBS (pH 7.4) and measured at 25 °C, with the infusion rate set at 50. Data acquisition involved the capture of five 60-second videos per sample at a camera level of 15. Analyses were performed using raw data algorithm and NTA software (version 3.4.003) with a detection threshold of five. Three independent measurements each with five technical replicates were performed for each sample.

***FI-AF4-MD* analysis** was performed using a flow field-flow fractionation system (Postnova Analytics, Germany) coupled with online multi-angle light scattering (MALS, PN3621), UV detection (260 nm, PN3242), and dynamic light scattering (Zetasizer Nano ZS, Malvern Panalytical). Separation was carried out using a frit-inlet (FI) channel with a 350 μm spacer and 10 kDa MWCO regenerated cellulose membranes. LNP samples (20 μL injection volume, 0.5 mg/mL) were injected using a 100 μL loop in triplicate. Elution was performed in PBS (pH 7.4) with an injection flow rate of 0.2 mL/min, a crossflow rate of 0.75 mL/min (exponential decay 0.2), and a detector flow rate of 0.3 mL/min. Data were processed using Nova FFF software (v2.2.0.1) applying the spherical MALS model (28–148°) for LNPs. Each LNP formulation was named according to the parameters in Table S3.

Transmission electron microscopy with negative staining was used to image LNP formulations for their morphology, in tandem with determination of the AF4 shape factor ratio. Copper grids with carbon film 400 were used (Agar Scientific, AGS160-4) and glow-discharged prior to sample application. A volume of 10 μL of LNP suspension was applied to the grid and incubated for 15 min. Excess sample was removed by filter paper adsorption, and the grid was rinsed once with dH2O, followed by a 5 min fixation with 2% glutaraldehyde. Samples were then washed three times with dH2O (30 s per wash) and 2% uranyl acetate applied for 5 min. Excess dye was removed by blotting using filter paper, and the grids

were left to dry at ambient temperature for 15 min. Samples were imaged using a JEM transmission electron microscope operated at 120 kV, at defined magnifications in Figure S 1.

Statistical Analysis

Tukey's one way ANOVA tests were utilised to determine statistically significant differences between different ASO-LNP formulation critical quality attributes, where *p<0.05, **p<0.005, ***p<0.0005. Comparative statistical analyses were carried out to correlate ASO-LNP formulation critical quality attributes. Statistical and correlative analyses were conducted using MiniTab v 20.4and data were graphed using OriginPro version 9.9.0.220.

ASO Analytics

Table S4 *IPRP-UV and HILIC-PDA percentage purity ratios of LMWS, FLP and HMWS of ON, (‡n=2 ± STD, #n=1).*

Sample	HILIC-PDA (%)			IP-RP-UV (%)		
	LMWS	FLP	HMWS	LMWS#	FLP#	HMWS#
ON	3.66 ± 0.08‡	95.34 ± 0.33‡	1.01 ± 0.25‡	8.05	83.76	7.68

ASO-LNP Analysis

Table S5 *DLS, ELS and RiboGreen Assay output CQAs per each ASO-LNP sample. PDI: polydispersity index; ZP: zeta potential; EE: encapsulation efficiency; MB: mass balance; FRR: flow rate ratio; TFR: total flow rate. n=3 (mean ± STD). Tukey Tests compared LNP1-2(*), LNP1-3(‡) and LNP3-4/5(#) where *p < 0.05, **p < 0.005 and ***p < 0.0005.*

Sample	Z-avgerage (d.nm)	Distribution Size (nm)	PDI	ZP (mV)	EE %	MB %
**SM102 LNP-1**<br>(N/P: 6, FRR 3, TFR 15)	76.7 ± 6.7	82.4 ± 7.0	0.14 ± 0.07	-1.2 ± 0.3	98.0 ± 0.4	54.4 ± 10.6
**SM102 LNP-2**<br>(**N/P: 12**, FRR 3, TFR 15)	77.4 ± 1.1	84.1 ± 1.2	0.08 ± 0.01	-1.3 ± 0.3	97.2 ± 0.3*	50.3 ± 3.7
**MC3 LNP-3**<br>(N/P: 6, FRR 3, TFR 15)	87.5 ± 1.2	92.4 ± 1.2	0.04 ± 0.00	-3.2 ± 0.4‡‡	98.2 ± 0.2	39.8 ± 3.9
**MC3 LNP-4**<br>(N/P: 6, **FRR 6**, TFR 15)	93.3 ± 1.1#	99.7 ± 1.8#	0.06 ± 0.01	-2.9 ± 0.2	97.4 ± 0.6	35.2 ± 9.8
**MC3 LNP-5**<br>(N/P: 6, FRR 3, **TFR 5**)	96.9 ± 1.9###	103.3 ± 2.8##	0.05 ± 0.01	-3.3 ± 0.4	97.0 ± 0.4#	43.8 ± 2.9

Table S6 *NTA mean, mode, 10th, 50th, and 90th centile diameters, and corresponding span values for ASO-LNP formulations, (n=3, mean ± STD). FRR: Flow Rate Ratio; TFR: Total Flow Rate. Tukey Tests compared LNP1-2(*), LNP1-3(‡) and LNP3-4/5(#) where $^{*}p < 0.05$, $^{**}p < 0.005$ and $^{***}p < 0.0005$.*

Sample	Mean (nm)	Mode (nm)	D10 (nm)	D50 (nm)	D90 (nm)	Span
**SM102 LNP-1**<br>**(N/P: 6**, FRR 3, TFR 15**)**	72.9 ± 1.6	66.3 ± 0.5	49.3 ± 0.6	69.6 ± 0.8	99.1 ± 3.9	0.72 ± 0.04
**SM102 LNP-2**<br>**(N/P: 12**, FRR 3, TFR 15**)**	74.4 ± 3.1	68.1 ± 3.9	52.8 ± 1.9*	70.3 ± 2.5	96.3 ± 4.3	0.62 ± 0.02*
**MC3 LNP-3**<br>(N/P: 6, FRR 3, TFR 15)	90.6 ± 1.1‡‡‡	83.5 ± 1.6‡‡‡	64.2 ± 1.1‡‡‡	87.4 ± 1.0‡‡‡	119.6 ± 0.7‡‡	0.63 ± 0.01‡
**MC3 LNP-4**<br>(N/P: 6, **FRR 6**, TFR 15)	92.5 ± 0.6	86.7 ± 1.9	63.2 ± 0.4	89.5 ± 0.6	124.1 ± 0.9$^{\#}$	0.68 ± 0.01$^{\#}$
**MC3 LNP 5**<br>(N/P: 6, FRR 3, **TFR 5**)	99.6 ± 2.2$^{\#\#}$	95.7 ± 5.5$^{\#}$	68.1 ± 1.2$^{\#}$	97.3 ± 2.3$^{\#\#\#}$	132.7 ± 2.5$^{\#\#\#}$	0.66 ± 0.01

NTA measurements showed similarities between mean and mode particle diameters for all LNP formulations, indicating minimal contributions from larger particles within each population, with a mean/mode size of ~80 nm (Table S 6). The small differences between mean and mode measured particle diameters suggest that spin-column purification may have removed a proportion of smaller-sized particles, increasing overall size uniformity within each sample. These observations were further supported by the span values (Table S 6), which followed the same trends as the PDI data. LNP-1 exhibited the broadest size distribution (0.72), whereas the remaining LNP formulations displayed narrower spans (0.62-0.68). Mode particle diameters increased progressively across samples by up to 30 nm, with LNP-5 formulations containing the largest particles (Table S 6). Distributional values (Figure 1, Table S 6) demonstrate, formulation and manufacture process impacts on LNP sub-population generation, with overall D10, D50 and D90 increased sizes with further investigating parameters.

FI-AF4-UV-MALS-DLS Evaluation of ASO-LNP Formulations

To perform further in-depth analysis of the ASO LNP formulations, AF4 was used to provide size-based separation of LNP particles based on their diffusion in the AF4 channel. For the purposes of this study, frit inlet AF4 was multiplexed with a UV detector for RNA quantification, MALS for measuring radius of gyration and molar mass approximation, and online DLS, for the measurement of hydrodynamic radius. Combining readout from the online MALS and DLS detectors, the shape factor was approximated, providing insights into LNP formulation morphology (R_G/R_H) and inherent structural differences between the LNP formulation.

Table S7 *Insights into FI-AF4-MD distributional values for R_G, R_H and shape factor elution profiles for ASO-LNP formulations. ET: Elution time; Rec: Recovery; FRR: Flow Rate Ratio; TFR: Total Flow Rate Tukey Tests compared LNP1-2(*), LNP1-3(‡) and LNP3-4/5(#) where *$p < 0.05$, **$p < 0.005$ and ***$p < 0.0005$, (n=3), error bars represent mean ± STD.*

Sample	FI-AF4-MD														
	Et (min)	Rec (%)	R_G Mode (nm)	R_G 10 (nm)	R_G 50 (nm)	R_G 90 (nm)	R_G Span	R_H 10 (nm)	R_H 50 (nm)	R_H 90 (nm)	R_H Span	SF 10	SF 50	SF 90	SF Span
**SM102 LNP-1 (N/P: 6, FRR 3, TFR 15)**	24.2 0.2	86.5 ± 8.5	24.7 ± 0.7	19.6 ± 1.5	22.1 ± 0.9	24.9 ± 1.6	0.24 ± 0.01	33.7 ± 0.3	36.5 ± 0.5	40.3 ± 1.0	0.18 ± 0.03	0.58 ± 0.04	0.60 ± 0.03	0.63 ± 0.04	0.08 ± 0.01
**SM102 LNP-2 (N/P: 12**, FRR 3, TFR 15)	23.7 ± 0.4	84.3 ± 1.2	26.7 ± 0.7	24.4 ± 0.8*	26.5 ± 0.6**	29.2 ± 1.1*	0.18 ± 0.02*	35.6 ± 0.4**	38.2 ± 0.4*	41.6 ± 0.5	0.16 ± 0.00	0.68 ± 0.01*	0.70 ± 0.01*	0.71 ± 0.01*	0.04 ± 0.01*
**MC3 LNP-3** (N/P: 6, FRR 3, TFR 15)	25.9 ± 0.5‡	88.9 ± 3.4	31.0 ± 0.8‡	27.3 ± 0.4‡‡	30.7 ± 0.6‡‡‡	35.0 ± 0.6‡‡‡	0.25 ± 0.02	38.3 ± 0.6‡‡‡	42.8 ± 0.5‡‡‡	47.6 ± 0.5‡‡‡	0.22 ± 0.01	0.71 ± 0.00‡	0.72 ± 0.00‡‡	0.74± 0.00‡	0.04 ± 0.01‡
**MC3 LNP-4** (N/P: 6, **FRR 6**, TFR 15)	26.7 ± 0.6	87.2 ± 2.4	33.4 ± 0.3$^{\#\#}$	26.1 ± 0.2	31.6 ± 0.6	37.4 ± 0.7$^{\#}$	0.36 ± 0.02$^{\#\#}$	40.1 ± 0.1	46.1 ± 1.0$^{\#}$	52.0 ± 1.1$^{\#\#}$	0.26 ± 0.02	0.65 ± 0.01$^{\#\#\#}$	0.69 ± 0.00$^{\#\#\#}$	0.72 ± 0.00$^{\#}$	0.10 ± 0.01$^{\#\#}$
**MC3 LNP 5** (N/P: 6, FRR 3, **TFR 5**)	27.9± 0.2$^{\#}$	85.8 ± 4.8	34.7 ± 0.8$^{\#\#\#}$	29.2 ± 0.2$^{\#\#}$	32.9 ± 0.9$^{\#}$	37.5 ± 0.9$^{\#}$	0.25 ± 0.01	42.8 ± 1.1$^{\#\#}$	47.1 ± 1.0$^{\#\#}$	52.3 ± 0.8$^{\#\#}$	0.23 ± 0.04	0.68 ± 0.00$^{\#\#}$	0.70 ± 0.01$^{\#\#}$	0.72 ± 0.01$^{\#}$	0.05 ± 0.01

Transmission Electron Microscopy

LNP formulations were imaged to further visualise differences between physicochemical attribute differences.

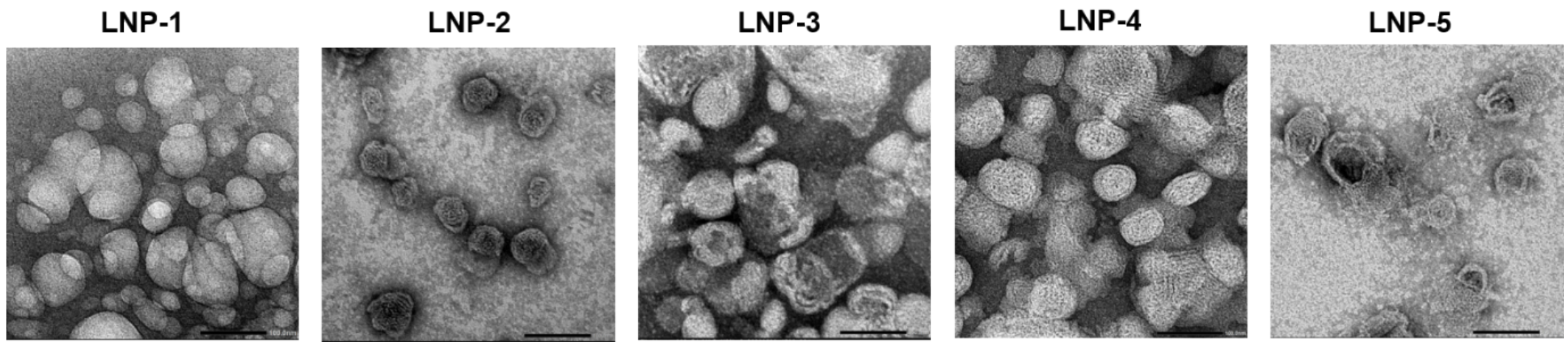

Figure S1 *Negative stain TEM images of ASO-LNP Formulations at 80k magnification (n=1).*

LNP formulations 1-5 produced variable results when imaged using negative stain electron microscopy.

ASO-LNP CQA Output Correlative Evaluations

By combining analytical technique CQA outputs, correlative impact between measured physicochemical attributes can be obtained, and LNP groupings were visualised to probe relationships between LNP formulations and their respective CQAs.

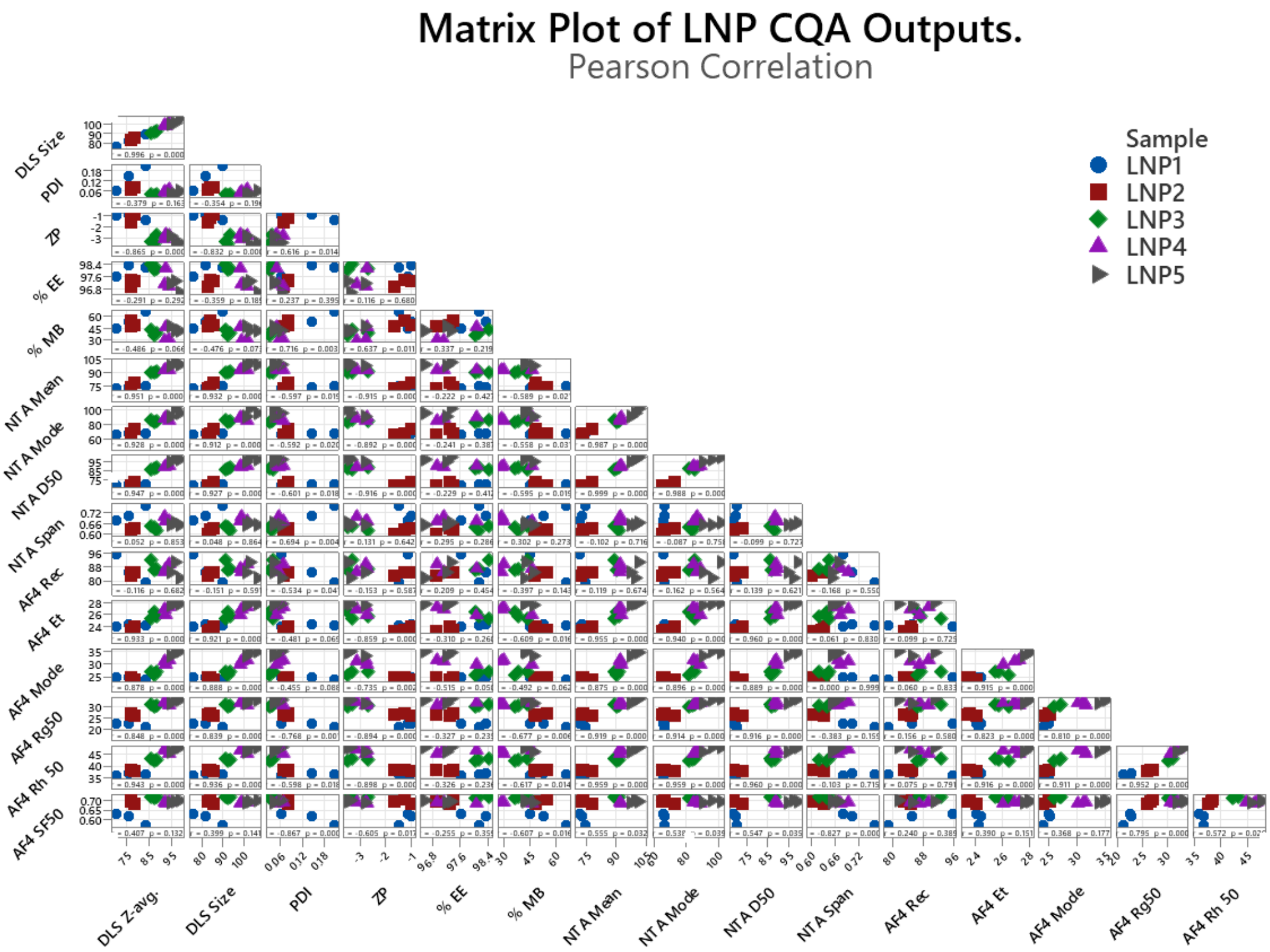

Figure S2 *Analytical technique CQA outputs sample grouping distribution between CQAs for ASO-LNPs.*

Correlation heatmaps provided further insight into relationships among CQAs across the tested LNP formulations (Figure S2). Mapping of the LNP samples revealed distinct clustering patterns based on comparable CQA profiles. Formulations LNP-1 and LNP-2, as well as LNP-3 to LNP-5, exhibited tighter clustering, suggesting that variations in N/P ratio, TFR, and FRR resulted in more incremental changes to CQA relationships than changes in ionisable lipid composition. These findings indicate that ionisable lipid selection exerts a greater influence on overall formulation characteristics, while optimisation of formulation parameters may enable finer tuning of CQA outputs. As our understanding of CQA interrelationships continues to improve, such analyses will support increasingly rational and data-driven LNP design and optimisation strategies (Figure S2).
